# Radiomics Nomogram in Assisting Lymphadenectomy Decisions by Predicting Lymph Node Metastasis in Early-Stage Endometrial Cancer

**DOI:** 10.3389/fonc.2022.894918

**Published:** 2022-05-31

**Authors:** Xue-Fei Liu, Bi-Cong Yan, Ying Li, Feng-Hua Ma, Jin-Wei Qiang

**Affiliations:** ^1^ Department of Radiology, Jinshan Hospital, Fudan University, Shanghai, China; ^2^ Department of Diagnostic and Interventional Radiology, Shanghai Jiao Tong University Affiliated Sixth People’s Hospital, Shanghai, China; ^3^ Departments of Radiology, Obstetrics & Gynecology Hospital, Fudan University, Shanghai, China

**Keywords:** endometrial cancer, early-stage, lymph node metastasis, radiomics nomogram, lymphadenectomy decision

## Abstract

**Background:**

Lymph node metastasis (LNM) is an important risk factor affecting treatment strategy and prognosis for endometrial cancer (EC) patients. A radiomics nomogram was established in assisting lymphadenectomy decisions preoperatively by predicting LNM status in early-stage EC patients.

**Methods:**

A total of 707 retrospective clinical early-stage EC patients were enrolled and randomly divided into a training cohort and a test cohort. Radiomics features were extracted from MR imaging. Three models were built, including a guideline-recommended clinical model (grade 1-2 endometrioid tumors by dilatation and curettage and less than 50% myometrial invasion on MRI without cervical infiltration), a radiomics model (selected radiomics features), and a radiomics nomogram model (combing the selected radiomics features, myometrial invasion on MRI, and cancer antigen 125). The predictive performance of the three models was assessed by the area under the receiver operating characteristic (ROC) curves (AUC). The clinical decision curves, net reclassification index (NRI), and total integrated discrimination index (IDI) based on the total included patients to assess the clinical benefit of the clinical model and the radiomics nomogram were calculated.

**Results:**

The predictive ability of the clinical model, the radiomics model, and the radiomics nomogram between LNM and non-LNM were 0.66 [95% CI: 0.55-0.77], 0.82 [95% CI: 0.74-0.90], and 0.85 [95% CI: 0.77-0.93] in the training cohort, and 0.67 [95% CI: 0.56-0.78], 0.81 [95% CI: 0.72-0.90], and 0.83 [95% CI: 0.74-0.92] in the test cohort, respectively. The decision curve analysis, NRI (1.06 [95% CI: 0.81-1.32]), and IDI (0.05 [95% CI: 0.03-0.07]) demonstrated the clinical usefulness of the radiomics nomogram.

**Conclusions:**

The predictive radiomics nomogram could be conveniently used for individualized prediction of LNM and assisting lymphadenectomy decisions in early-stage EC patients.

## Introduction

Endometrial cancer (EC) is the most common gynecologic malignancy in industrialized countries (1). Tumor size, tumor grade, histological subtype, depth of myometrial invasion (MI), lymphovascular space invasion (LVSI), and lymph node metastasis (LNM) are known prognostic factors of EC (2). According to the International Federation of Gynecology and Obstetrics, complete pelvic and para-aortic lymphadenectomy was the recommended surgical treatment for stage II-IV EC patients (3). However, the therapeutic value of lymphadenectomy in early-stage EC is still in debate, as no improvement in disease-free survival or overall survival (OS) was found in early-stage EC with or without lymphadenectomy (4).

Lymphadenectomy is not recommended by the Gynecologic Oncology Group (GOG) in early-stage EC patients with grade 1 or 2 and superficial MI (<50% MI) (5). Furthermore, based on a landmark GOG-33 staging study, an overall 9% risk of LNM was reported in clinical early-stage EC (6). In addition, lymphadenectomy resulted in longer operating times, more blood loss, higher transfusion rates, and longer hospital stays (7). Thus, preoperative evaluation of early-stage EC is clinically useful in helping with lymphadenectomy decion-making for these patients.

Magnetic resonance imaging (MRI) is a useful tool which allows for noninvasive visualization of anatomic structures with high spatial resolution and soft tissue contrast. However, a meta-analysis indicated that MRI has low sensitivity and specificity in diagnosing LNM in EC patients (8). Metastasis in a normal-sized lymph node (LN) can be missed, and inflammatory LN enlargement cannot be reliably distinguished from LNM by conventional MRI (9). Radiomics, a method of high-throughput extraction of quantitative medical image features, might improve standard visual image analysis and offer valuable information for diagnostic and prognostic purposes (10). A previous study showed that MRI-based radiomics are efficient in helping the radiologists in identifying LNM preoperatively (9). In addition, a radiomics nomogram combining the radiomics features and clinical risk factors could be conveniently applied to help clinical management decisions (11).

We assumed that the radiomics nomogram could be a useful tool in helping clinical management decisions in early-stage EC. Thus, the aim of this study was to develop and validate a clinical- and radiomics-based nomogram for the preoperative prediction of LNM individually in assisting lymphadenectomy decisions in patients with early-stage EC.

## Materials and Methods

### Patients

**Figure 1:**
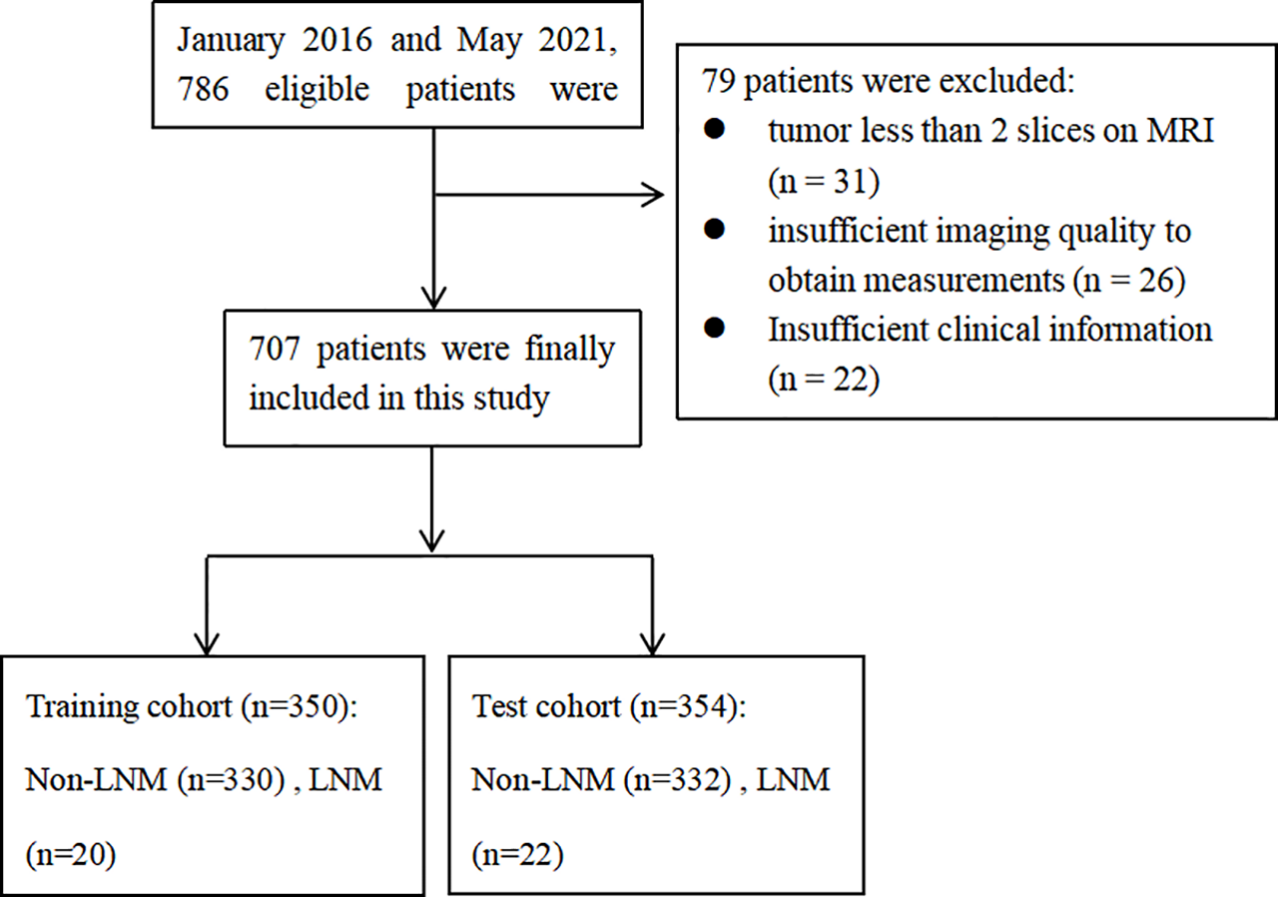
The workflow of this study.

This retrospective study was reviewed and approved by the Institutional Review Board of Obstetrics & Gynecology Hospital of Fudan University (No. 2020-10). All patients signed the informed consent. In total, 707 patients from January 2016 to May 2021 were included in this study. All patients met the following inclusion criteria (1): histopathology confirmed EC and pelvic/aortic LNM status (2); patients underwent a dilatation and curettage (D&C); (3) patients underwent MRI planning including T1-weighted imaging (T1WI), T2-weighted imaging (T2WI), diffusion-weighted imaging (DWI), apparent diffusion coefficient (ADC) maps, and contrast-enhanced (CE) T1WI within 30 days before the surgery. Patients were excluded if: (1) tumor less than 2 slices on the MRI scan; (2) insufficient imaging quality to obtain measurements or insufficient clinical information; (3) cervical infiltration or extra-uterine tumor showed on MRI. The flow chart of inclusion and exclusion criteria is shown in **Figure 1**.

The 707 patients were randomly divided into a training cohort and a test cohort, according to the ratio of 5:5. Clinical information of all patients were obtained from the medical records, including D&C tumor grade, MRI-reported MI status, age, metabolic syndrome, and cancer antigen 125 (CA125). Patients with pelvic LN > 8 mm or abdominal LN > 10 mm, or with non-homogeneous enhancement and central necrosis on CE-T1WI images were considered as MR-report LNM positive (12). For the patients with total hysterectomy and bilateral salpingo-oophorectomy without lymphadenectomy, the follow-up of at least two years was used to confirm if the patient had LNM or not.

### Development of the Clinical Model

The clinical model for preoperatively deciding whether a patient required lymphadenectomy was built according to the recommendation from the Society of Gynecologic Oncology Clinical Practice EC Working Group (5). Patients with grade 1-2 endometrioid tumors (by D&C), less than 50% MI (on MRI without cervical infiltration), and tumor of 2 cm or less require no lymphadenectomy.

### MRI Acquisition and Segmentation

MRI was performed using a 1.5-T MR system (Magnetom Avanto, Siemens, Germany). The following sequences were obtained: axial spin-echo (SE) T1-weighted imaging (T1WI) with repetition time (TR)/echo time (TE) = 761/10 ms; turbo axial SE T2-weighted imaging (T2WI) with fat saturation (TR/TE = 4000/98 ms); sagittal T2WI (TR/TE = 3849/83 ms) and coronal T2WI (TR/TE = 4490/83 ms); Axial echo planar imaging DWI was obtained with b values of 0 and 800 s/mm^2^. Sagittal and coronal CE-T1WI with FS (TR/TE =439/10 ms, thickness = 4 mm) and axial CE-T1WI with FS (TR/TE = 196/2.9 ms, thickness = 4 mm) were performed at the arterial phase (30-40 sec), venous phase (75-90 sec), and delayed phase (120-180 sec) after the intravenous administration of gadopentetate dimeglumine (Magnevist, Bayer Schering, Berlin, Germany) at a dose of 0.2 mmol/kg of body weight and a rate of 2 to 3 mL/s.

Tumor segmentation was performed by manually delineating (by radiologist 1) the region of interest (ROI) along the tumor contour on each axial T2WI and then referred to the DWI (the tumor area showed as high signal in high b value sequences), ADC (the tumor area showed as low signal), and axial DCE images (delayed phase) using an open-source imaging platform (MITK, version 4.9.0; http://www.mitk.org). Thirty days later, 50 randomly chosen images were used to assess the reliability for each radiomics feature. The ROI delineation was performed separately by two radiologists (radiologist 1 and radiologist 2, with 3 and 11 years of experience in pelvic imaging, respectively). The radiologists were blinded to the clinical and histopathology information. The reliability was calculated using the intraclass/interclass correlation coefficient (ICC). The features with ICCs greater than 0.75 indicated satisfactory reproducibility of radiomics feature extraction and were retained.

### Radiomics Features Extraction and Selection

All feature extractions were implemented in Pyradiomics package of Python (v.3.9; https://www.python.org) The radiomics features, including shape-based, first-order, and texture features were extracted. Pearson’s correlation was used to identify redundant features. If two features had a Pearson correlation coefficient > 0.9, the one with larger mean absolute coefficient was eliminated. Synthetic minority oversampling technique (SMOTE) method was used because of the unbalance of positive/negative LNM samples in the training cohort. Positive LNM (minority class) were over-sampled and negative LNM (majority class) were under-sampled to balance the training cohort to improve the classification performance. Then, a least absolute shrinkage and selection operator (LASSO) regression with 10-fold cross-validation was used to obtain the most significant features (radiomics signatures) for predicting LNM in the training cohort.

### Clinical Risk Factors Selection

A multivariate logistic regression analysis was used to identify the clinical independent risk factors (age, metabolic syndrome, tumor size, MRI-reported MI, and CA125) for LNM in the training cohort. Backward stepwise selection was applied. The stopping rule was that the likelihood ratio test achieved a least Akaike’s information criterion.

### Development and Validation of the Radiomics Model, the Radiomics Nomogram Model, and the Clinical Model

A radscore was calculated for each patient from the training cohort *via* a linear combination of radiomics signatures that were weighted by their respective coefficients.

A multivariate logistic regression was applied to build the radiomics nomogram, which can be used as a visualized and individual tool that integrated the radiomics signatures with independent clinical risk factors to predict the probability of LNM in the training cohort.

The area under the curve (AUC) of the receiver operating characteristic (ROC) curve was calculated to assess the predictive performance of the three models. The sensitivity, specificity, and AUC of the nomogram were calculated in the training cohort and validated in the test cohort.

### Clinical Usefulness Analysis

Decision curve analysis was conducted to determine the clinical usefulness of the radiomics nomogram and the clinical model by quantifying the net benefits at different threshold probabilities using the training and test cohorts.

The performances of the radiomics nomogram and the clinical model were compared using net reclassification index (NRI) and total integrated discrimination index (IDI) by using the entire dataset.

### Statistical Analysis

Statistical analysis was performed using R software (version 4.05; http://www.Rproject.org). Independent sample t-test (if met normality and variance homogeneity) or Mann-Whitney U (if not met normality or variance homogeneity) were used to compare the differences in continuous variables (age, CA125, tumor size, and radscore) between the LNM and non-LNM patients; and the chi-squared test was used to compare the differences in categorical variables (metabolic syndrome, D&C-reported tumor grade, MRI-reported MI, and histopathology-reported tumor grade, MI, LVSI, and histological subtype) between the LNM and non-LNM patients in both the training and test cohorts. Association between the radiomics signatures and clinical risk factors was further assessed using Spearman’s correlation. The “glmnet” package was used for LASSO and logistic regression, the “DMwR” package was used for SMOTE, the “rms” package was used for nomogram calculation, the “pROC” package was used for AUC, and the “dca.R” package was used for DCA. ROC curve analysis was performed to calculate the AUC and corresponding 95% confidence interval. A P value less than 0.05 was considered statistically significant.

## Results

### Clinical Characteristics

Among the 707 patients (aged 55 ± 8.9, ranged 25-89) with early-stage EC, 42/665 patients had LNM/non-LNM. Sixty-five patients had total hysterectomy and bilateral salpingo-oophorectomy without lymphadenectomy, none of them were found LNM within 2 years follow-up. These patients were considered as non-LNM. The clinicopathologic characteristics of all the patients are shown in **Table 1**.

**Table 1:** The comparisons of clinicopathologic characteristics between LNM and non-LNM patients in training and test cohorts.

	Training cohort			Test cohort		
	non-LNM (N=333)	LNM (N=20)	P-value	non-LNM (N=332)	LNM (N=22)	P-value
Radscore	0.052 (0.062)	0.133 (0.071)	<0.001	0.057 (0.065)	0.137 (0.068)	<0.001
CA125	23.8 (20.1)	71.1 (83.7)	0.021	24.3 (23.3)	44.5 (45.8)	0.052
Age	55.9 (9.1)	54.9 (8.3)	0.580	55.3 (8.9)	56.8 (8.3)	0.424
Tumor size	17.1 (6.8)	24.1 (12.2)	0.019	16.4 (6.5)	21.7 (8.3)	0.008
Metabolic syndrome			0.450			0.117
(–)	171 (51.4%)	8 (40.0%)		171 (51.5%)	7 (31.8%)	
(+)	162 (48.6%)	12 (60.0%)		161 (48.5%)	15 (68.2%)	
D&C tumor grade			0.357			1
G1	284 (85.3%)	15 (75.0%)		288 (86.7%)	19 (86.4%)	
G2	49 (14.7%)	5 (25.0%)		44 (13.3%)	3 (13.6%)	
MRI MI			0.042			<0.001
(-)	294 (88.3%)	14 (70.0%)		297 (89.5%)	13 (59.1%)	
(+)	39 (11.7%)	6 (30.0%)		35 (10.5%)	9 (40.9%)	
MRI LM			1			0.477
(-)	323 (97.0%)	19 (95.0%)		320 (96.4%)	20 (90.9%)	
(+)	10 (3.0%)	1 (5.0%)		12 (3.6%)	2 (9.1%)	
Clinical decision lymphadenectomy			0.010			0.002
(-)	220 (66.1%)	7 (35.0%)		234 (70.5%)	8 (36.4%)	
(+)	113 (33.9%)	13 (65.0%)		98 (29.5%)	14 (63.6%)	
Histopathology tumor grade			0.059			<0.001
AH	1 (0.3%)	0 (0%)		0 (0%)	0 (0%)	
G1	232 (69.7%)	13 (65.0%)		228 (68.7%)	8 (36.4%)	
G2	85 (25.5%)	4 (20.0%)		81 (24.4%)	9 (40.9%)	
G3	11 (3.3%)	1 (5.0%)		15 (4.5%)	1 (4.5%)	
Non-endometrioid	4 (1.2%)	2 (10.0%)		8 (2.4%)	4 (18.2%)	
Histopathology MI			0.040			<0.001
Non-MI	83 (24.9%)	3 (15.0%)		97 (29.2%)	0 (0%)	
Superficial MI	192 (57.7%)	9 (45.0%)		184 (55.4%)	10 (45.5%)	
Deep MI	58 (17.4%)	8 (40.0%)		51 (15.4%)	12 (54.5%)	
LVSI			<0.001			<0.001
(-)	292 (87.7%)	7 (35.0%)		282 (84.9%)	5 (22.7%)	
(+)	41 (12.3%)	13 (65.0%)		50 (15.1%)	17 (77.3%)	
Histopathology tumor type			0.008			<0.001
Endometrioid Adenocarcinoma	328 (98.5%)	18 (90.0%)		324 (97.6%)	18 (81.8%)	
Mixed Adenocarcinoma	2 (0.6%)	1 (5.0%)		4 (1.2%)	2 (9.1%)	
Serous Adenocarcinoma	1 (0.3%)	1 (5.0%)		3 (0.9%)	2 (9.1%)	
Other	2 (0.6%)	0 (0%)		1 (0.3%)	0 (0%)	

AH, atypical hyperplasia; CA125, cancer antigen 125; D&C, dilatation and curettage; LVSI, lymphovascular space invasion; MI, myometrial invasion.

The time interval between D&C and MR scanning is 5.8 ± 2.4 d, ranged 0-10d (0, on the same day). Tumor grade (D&C diagnosed G1 and G2) and MI status (MRI diagnosed MI status) were downgraded in 92 cases after surgery. Of these, 30 (4.2%) were downgraded from G1/G2 to AH/G1; 62 (8.7%) were downgraded from DMI to SMI/non-MI, respectively. On the contrary, 46 (6.5%) were upgraded from G1/G2 to G3/non-endometrioid adenocarcinoma; 102 (14.4%) were upgraded from non-MI/SMI to DMI according to the final pathology examination, respectively.

### Development of the Clinical Model

According to the clinical model, 469 (66.3%) patients were identified as ineligible candidates for lymphadenectomy and 238 (33.7%) patients were identified as eligible candidates for lymphadenectomy (**Table 1**).

### Radiomics Features Selection and Radiomics Signatures Construction

**Figure 2:**
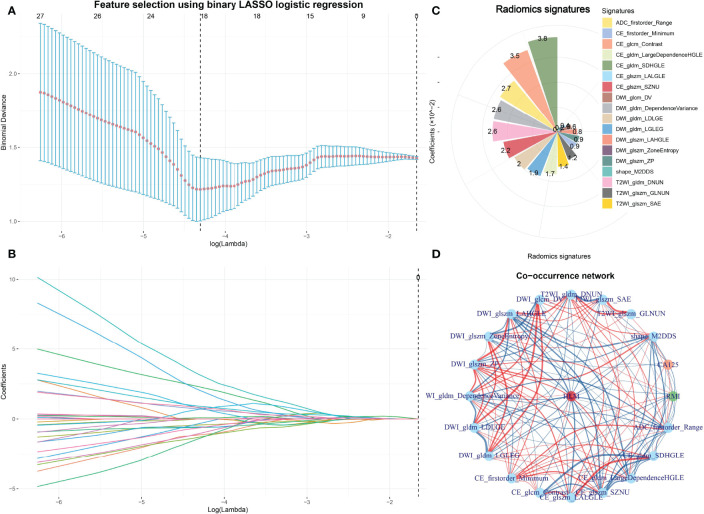
Feature selection using LASSO and the selected radiomics signatures and co-occurrence of radiomics signatures and clinical features. The parameter lambda is chosen using 10-fold cross-validation *via* minimum criteria, which resulted in 10 features with nonzero coefficients **(A)**. LASSO coefficient profiles of the selected features **(B)**. The selected radiomics signatures of LNM by the LASSO method **(C)**. A co-occurrence map shows the correlations between radiomics features and clinical features of LNM in early-stage EC **(D)**.

**Figure 3:**
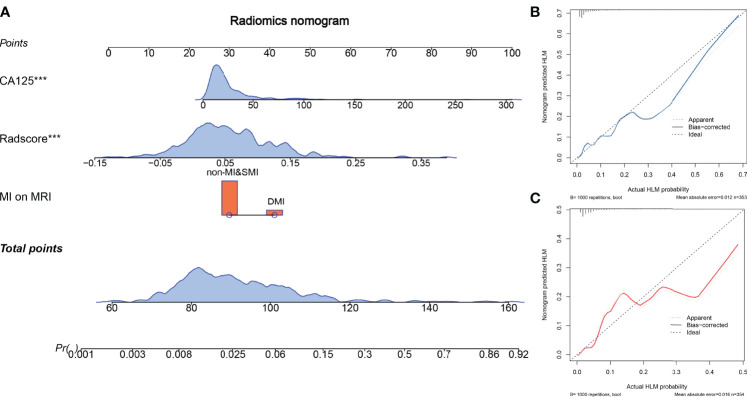
The radiomics nomogram and calibration curves. The radiomics nomogram is constructed by integrating CA125, radscore, and myometrial invasion (MI) on MRI **(A)**. Calibration curve of the radiomics nomogram for predicting LNM in the training cohort **(B)** and the test cohort **(C)**.

In the training cohort, 358 features were extracted from the T1WI, T2WI, DWI, CE, and ADC images. After removing features with either interobserver or intraobserver ICC < 0.75 and Pearson correlation coefficients > 0.9, 234 and 89 features were retained, respectively. LASSO analysis finally included 18 radiomics features, which were defined as the radiomics signatures (**Figure 2** and **Figure 3**). The association between radiomics signatures and clinical risk factors is shown in **Figure 3**. The radscore calculation is shown in the following: Radscore = 0.04994 + 0.0017×shape_M2DDS + -0.01212×T2WI_glszm_GLNUN + 0.01356×T2WI_glszm_SAE + -0.02575×T2WI_gldm_DNUN + 0.00589×DWI_glcm_DV + -0.00816×DWI_glszm_LAHGLE + -0.00132×DWI_glszm_ZoneEntropy + -0.00855×DWI_glszm_ZP + 0.02578×DWI_gldm_DependenceVariance + -0.01982×DWI_gldm_LDLGE + 0.01929×DWI_gldm_LGLEG + -0.00855×CE_firstorder_Minimum + -0.03477×CE_glcm_Contrast + -0.00393×CE_glszm_LALGLE + 0.0224×CE_glszm_SZNU + 0.01677×CE_gldm_LargeDependenceHGLE + 0.03809×CE_gldm_SDHGLE + -0.027×ADC_firstorder_Range

### Radiomics Nomogram Development and Validation

**Figure 4:**
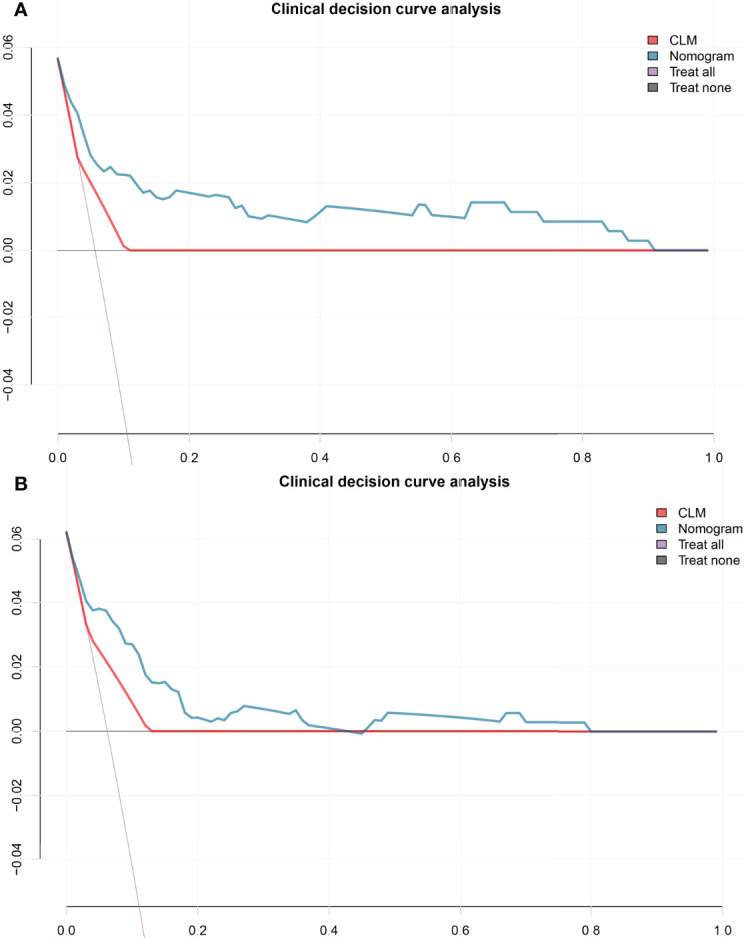
The decision curve shows that when the threshold probability from 10% to 90%, the radiomics nomogram adds more net benefit than schemes of treat-all, treat-none and radscore in the training cohort **(A)**, and the decision curve of the test cohort **(B)**. CLM, clinical model.

Multivariate logistic regression analysis showed that CA125 and tumor size were the risk factors for LNM in the early stage of EC. Considering that the selected feature “shape_M2DDC” reflects the tumor size, we did not include tumor size in the nomogram for avoiding over-fitting. Therefore, the radiomics nomogram was constructed by integrating the CA125, radscore, and MRI-reported MI status (**Figure 4**). The ROC curves of the three models in the training and test cohorts are shown in **Table 2**.

**Table 2:** Diagnostic performance of clinical model, radscore, and radiomics nomogram in the training and test cohorts.

Cohort	Index	AUC	95% CI	SPE	SEN	NPV	PPV	P*	P^#^
Training	Clinical model	0.66	0.55-0.77	0.66	0.65	0.97	0.10	0.004	–
	Radscore	0.82	0.74-0.90	0.80	0.75	0.98	0.18	–	0.004
	Nomogram	0.85	0.77-0.93	0.64	0.95	1.00	0.14	0.306	< 0.001
Test	Clinical model	0.67	0.56-0.78	0.70	0.64	0.97	0.13	0.005	–
	Radscore	0.81	0.72-0.90	0.56	0.95	0.99	0.13	–	0.005
	Nomogram	0.83	0.74-0.92	0.84	0.77	0.98	0.24	0.302	< 0.001

AUC, area under the curve; CI, confidence interval; NPV, negative predictive value; PPV, positive predictive value; SEN, sensitivity; SPE, specificity.

*Compared with Radscore; ^#^Compared with clinical model by Delong test.

### Clinical Usefulness

The decision curve analysis indicated that when the threshold probability was within a range from 10% to 90%, the net benefit of using the nomogram to predict LNM was greater than that of the treat-all or treat-none scheme in the training and test cohorts (**Figure 4**).

**Figure 5:**
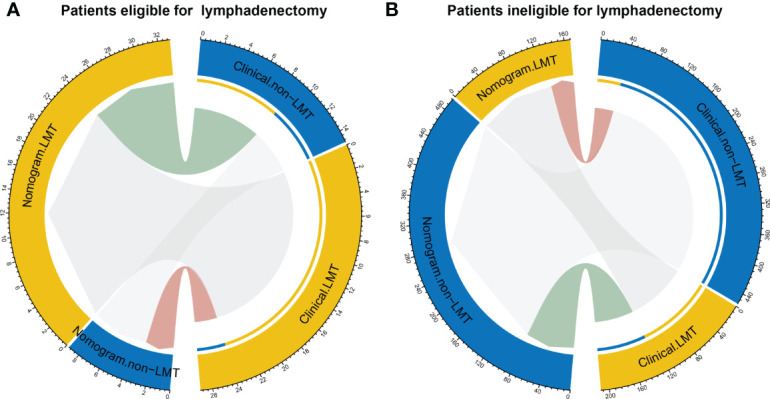
Reclassification of patients for eligible for lymphadenectomy **(A)** and in eligible for lymphadenectomy (LMT) **(B)**. Groups are illustrated according to the radiomics nomogram and clinical model-determined lymphadenectomy eligibility basing on the entire dataset with the specific patient numbers are presented. The patients were pathological confirmed whether eligible for lymphadenectomy. In the circle plots, the patients who were classified both correctly by clinical and nomogram are represented as connections in light grey. The connections in light green indicate patients who were clinically diagnosed incorrectly but reclassified correctly by the nomogram, while connections in pink indicate patients who were clinically diagnosed correctly but reclassified incorrectly by the nomogram.

The reclassification measures of discrimination confirmed that the radiomics nomogram performed better than the clinical model based on entire dataset with an NRI of 1.06 (95% confidence interval [CI]: 0.81-1.32) and an IDI of 0.05 (95% CI: 0.03-0.07) (both P < 0.001). Eighty-two patients were misclassified by the clinical model as candidates eligible for lymphadenectomy and 39 of them were corrected by the radiomics nomogram reclassification. Nine patients were misclassified by the clinical model as candidates ineligible for lymphadenectomy and three of them were corrected by the radiomics nomogram reclassification (**Figure 5**).

## Discussion

In this study, a preoperative individualized radiomics nomogram was developed and validated for predicting LNM in early-stage EC. The nomogram incorporated the radiomics signatures with two preoperative clinical risk factors (CA125 and MRI-reported MI status). This radiomics nomogram exhibited a good ability to predict LNM both in the training and test cohorts, which was easy to use and facilitated the preoperative individualized lymphadenectomy decision-making in early-stage EC.

Preoperatively assessing LNM status is crucial to guide the surgical management for EC patients. D&C and MRI are two recommended ways to preoperatively evaluate the tumor histological subtype, tumor grade, depth of MI, parametrial infiltration, and LNM (2). However, there is a relatively frequent discordance between the findings of D&C and final surgical pathology (12). Helpman et al. reported that 22% of G1 EC diagnosed by biopsy were upgraded to G2 or G3 in the final surgical pathology. As is shown in the result, 12.9% G1/G2 EC diagnosed by D&C were misdiagnosed. Furthermore, the approach of intra-operative frozen section is not readily available in most major cancer centers (13). However, a previous study showed that the radiomics features combining with ADC value could be used effectively to evaluate tumor grade with a AUC of 0.95 (14).

A previous study showed that conventional MRI is limited in detecting metastatic LN, even when the radiologists were informed of the radiomics prediction results of LN status (9). The reason for this disadvantage might be attributed to the metastatic LN having a normal size (< 0.8 cm), morphology, signal, or due to the MRI partial volume effects (9). However, the MRI-based radiomics model could be used to assess the LN status and help radiologists improve their performance in predicting LNM in EC (9). In accordance with the previous study, results showed a good ability to predict LNM both in the training and test cohorts in this study.

CA125 was found to be an independent risk factor for LNM in early-stage EC. CA125 is also a risk factor for high-risk EC (15). Several guidelines, including the European Society of Medical Oncology, European Society of Gynecological Oncology, and European Society for Radiotherapy and Oncology (ESMO-ESGO-ESTRO) consensus conference guideline, incorporate measurement of CA125 to assess LN status along with imaging as part of preoperative workup (3). The tumor size is also commonly used as a prognostic factor in EC, since it has been correlated with LN status and prognosis in EC patients (16).

A previous study reported a 5%-9% risk of LNM in G1 or G2 endometrioid endometrial carcinoma, which means when classifying patients based on preoperative histology, a substantial number of patients with LNM will be missed (17). In early-stage EC patients, 5.9% were found to have LNM in this study, which is in accordance with the previous report. Furthermore, it is estimated that 33% of patients with a preoperative histological diagnosis of non-LNM are upgraded to LNM on final postoperative histological examination, resulting in an incorrect risk estimation of LNM (18). However, with the help of the radiomics nomogram, selective lymphadenectomy approaches might prevent unnecessary lymphadenectomy in low-risk (defined as G1 or G2 endometrioid endometrial carcinoma with MI less than 50% and primary tumor diameter less than 2 cm) patients.

The standard treatment of early-stage EC is hysterectomy and bilateral salpingo-oophorectomy (BSO), which may be performed *via* a laparotomy or by a laparoscopic approach (19). For patients with advanced stage EC (tumor spread beyond the womb), adjuvant radiotherapy (and increasingly chemotherapy) is administered to reduce the risk of recurrence. A previous study reported that no significant differences in 5-year survival rates were shown in patients with stage I and II disease who did or did not undergo lymphadenectomy (19). Furthermore, lymphadenectomy may not be routinely performed, and if it is, the extent of lymphadenectomy can range from taking a few LNs for sampling to performing complete dissection pelvic and para-aortic lymphadenectomy (19). In this study, LNM could be predicted by the radiomics nomogram, which would be used to reduce unnecessary morbidity caused by extensive LN dissection and improve staging by targeted removal of metastatic LNs missed by preoperative MRI scanning in early-stage EC. Studies assessing the diagnostic accuracy of sentinel LN biopsy have yielded promising results in the management of EC (20). Radiomics is expected to have the ability to predict and identify a metastatic LN, so as to further enhance the accuracy of sentinel LN biopsy.

Our study had several limitations. First, the current study included an inherent shortcoming by retrospective analysis of the patient records and the radiomics nomogram was established on the basis of single-center data. The robustness and reproducibility of the radiomics nomogram need to be further validated in prospective multi-center studies with larger participant pools. Second, the training cohort is re-sampled before constructing the radiomics nomogram. Bias might exist due to the imbalance of the samples. Third, all the enrolled patients received D&C before pelvic MRI scanning. D&C may result in decreased tumor volume, leading to some small tumors to be invisible on MRI. However, these cases were excluded from this study. Furthermore, the tumor size is positively correlated with lymph node metastasis in early-stage EC patients. D&C may lead to underestimates of lymph node metastasis. Further studies are warranted to investigate the effect on MRI after curettage by comparing MRI findings before and after curettage. Last, more studies that focus on comparing radiomics and prospective and randomized preoperative predictive techniques in systematic lymphadenectomy should be carried out.

In conclusion, we developed a convenient radiomics nomogram model that combines the D&C-reported tumor grade, MRI-reported MI status, clinical risk factors, and radiomics signatures (radscore) to preoperatively and non-invasively evaluate LN status in patients with early-stage EC. The application of the radiomics model could optimize clinical decision-making and potentially improve the selection of surgical scheme of early-stage EC patients.

## Data Availability Statement

The datasets presented in this study can be found in online repositories. The names of the repository/repositories and accession number(s) can be found below: The data is available at https://gitee.com/dr_yingli/EC_LM.

## Ethics Statement

The studies involving human participants were reviewed and approved by Ethics Committee of Jinshan Hospital, Fudan University (No. JIEC 2021-S55). The patients/participants provided their written informed consent to participate in this study.

## Author Contributions

B-CY and F-HM designed the research study. X-FL, B-CY, and YL performed the research. B-CY and F-HM provided help and advice on acquisition of data. YL and X-FL analyzed the data. YL and X-FL wrote the manuscript. YL and J-WQ were the supervisors of this study. All authors read and approved the final manuscript.

## Funding

This research was funded by Shanghai Science and Technology Committee (No. 20JC1418200) and National Natural Science Foundation of China (No. 81971579).

## Conflict of Interest

The authors declare that the research was conducted in the absence of any commercial or financial relationships that could be construed as a potential conflict of interest.

## Publisher’s Note

All claims expressed in this article are solely those of the authors and do not necessarily represent those of their affiliated organizations, or those of the publisher, the editors and the reviewers. Any product that may be evaluated in this article, or claim that may be made by its manufacturer, is not guaranteed or endorsed by the publisher.
